# Analysis of Cardiac Amyloidosis Progression Using Model-Based Markers

**DOI:** 10.3389/fphys.2020.00324

**Published:** 2020-04-30

**Authors:** Wenguang Li, Alan Lazarus, Hao Gao, Ana Martinez-Naharro, Marianna Fontana, Philip Hawkins, Swethajit Biswas, Robert Janiczek, Jennifer Cox, Colin Berry, Dirk Husmeier, Xiaoyu Luo

**Affiliations:** ^1^School of Mathematics and Statistics, University of Glasgow, Glasgow, United Kingdom; ^2^Centre for Amyloidosis and Acute Phase Proteins, University College London, London, United Kingdom; ^3^GlaxoSmithKline plc, Stevenage, United Kingdom; ^4^British Heart Foundation Glasgow Cardiovascular Research Centre, University of Glasgow, Glasgow, United Kingdom

**Keywords:** cardiac amyloidosis, left ventricle, model-based markers, classification, strain and stress, shape analysis, MRI

## Abstract

Deposition of amyloid in the heart can lead to cardiac dilation and impair its pumping ability. This ultimately leads to heart failure with worsening symptoms of breathlessness and fatigue due to the progressive loss of elasticity of the myocardium. Biomarkers linked to the clinical deterioration can be crucial in developing effective treatments. However, to date the progression of cardiac amyloidosis is poorly characterized. There is an urgent need to identify key predictors for disease progression and cardiac tissue function. In this proof of concept study, we estimate a group of new markers based on mathematical models of the left ventricle derived from routine clinical magnetic resonance imaging and follow-up scans from the National Amyloidosis Center at the Royal Free in London. Using mechanical modeling and statistical classification, we show that it is possible to predict disease progression. Our predictions agree with clinical assessments in a double-blind test in six out of the seven sample cases studied. Importantly, we find that multiple factors need to be used in the classification, which includes mechanical, geometrical and shape features. No single marker can yield reliable prediction given the complexity of the growth and remodeling process of diseased hearts undergoing high-dimensional shape changes. Our approach is promising in terms of clinical translation but the results presented should be interpreted with caution due to the small sample size.

## 1. Introduction

Amyloidosis occurs when proteins that take abnormal forms known as amyloid deposits build up in the tissues. These deposits are composed of abnormal protein fibers that accumulate more quickly than they are cleared away, and thus interfere with the structure and function of affected organs throughout the body. These include the heart, liver, skin, lungs, kidneys, and nervous system (Gertz et al., 2013). When amyloid fibrils infiltrate in myocardium, the ventricles will show impaired contraction and relaxation. This is known as cardiac amyloidosis. The most prevalent forms of cardiac amyloidosis are known as Transthyretin-related (ATTR) and immunoglobulin light chain (AL) amyloidosis (formerly known as primary amyloidosis). Untreated cardiac amyloid, particularly the AL type, can be life-threatening, the median survival of patients is half a year from the onset of heart failure (Grogan et al., 2017).

The left ventricle (LV) with amyloid becomes firm, rubbery and stiff, similar to hypertrophic cardiomyopathy (Kholova and Niessen, 2005). Further, the ventricular wall is thickened (Carroll et al., 1982; Kholova and Niessen, 2005; Quarta et al., 2012; Martinez-NaharrO et al., 2018), particularly in the interventricular septum (Frenzel et al., 1986), but the ventricular cavity does not dilate much (Kholova and Niessen, 2005). Hence, the functional defect in amyloidosis is associated to the “stiff heart” syndrome, with the LV end-diastolic pressure rising to at least 10 mmHg higher than normal subjects (Chew et al., 1975; Swanton et al., 1977). As a result, the amyloidosis myocardium material properties also altered (Petre et al., 2005).

With effective treatments, it is hoped that amyloid deposits can gradually diminish in patients. However, although various anti-amyloid drugs are being researched, none has been introduced into routine clinical practice. A standing challenge in developing anti-amyloid drugs is the difficulty of reliably assessing the disease progression non-invasively and within in a short follow-up duration, because subtle changes inside tissues with reduced amyloid deposits are not always visible in clinical images, such as cardiac magnetic resonance (CMR) imaging.

Using Doppler echocardiography, Koyama et al. found that the early impairment in systolic function of a cardiac amyloidosis heart can be reflected by changed longitudinal strain and strain rate (Koyama et al., 2003). Both circumferential and longitudinal strains are found to be substantially lower in an amyloidosis LV, compared with a normal, or hypertrophic cardiomyopathy LV (Sun et al., 2009; Buss et al., 2012). CMR images are used to diagnose cardiac amyloid with late gadolinium enhancement (LGE) (Vogelsberg et al., 2008; Liu et al., 2013; Dungu et al., 2014) or delayed enhancement (White et al., 2014). Based on CMR basal and apical short-axis images, White et al. showed that the peak LV twist rate and untwist rates are significantly lower in patients with cardiac amyloid LV (White et al., 2014). Nucifora et al. (2014) measured the circumferential strain of 61 amyloidosis patients using tagging CMR and found the peak circumferential strain could be a potential clinical biomarker.

In previous studies, strain and material stiffness were found to be associated with cardiac amyloidosis. However, there is much to be done on understanding the disease progression of cardiac amyloid. Needless to say, finding a reliable classification based on suitable biomarkers is crucially important in assessing the effectiveness of amyloidosis treatments and any clinical trials for new drugs. Despite major research development of computational cardiac models, which can provide a rich set of biomarkers, it is perhaps surprising that very little modeling effort has focused on cardiac amyloidosis (Chapelle et al., 2015), and no studies considered the relation to amyloidosis disease progression.

The aim of this work is to carry out an image-derived mechanical and statistical modeling approach for LVs with amyloidosis progression. We systemically checked multiple factors, including the strains, stresses, p-V curve, LV shape, and volume of a group of amyloidosis patients before and after treatment. The biomechanical modeling analysis was blind to the clinical assessment, and the classification based on the multiple factors compares favorably with the clinical observation. To the best of the authors' knowledge, this is the first time that cardiac amyloidosis progression has been studied in a combined mechanical and statistical approach, based on longitudinal images of real patients during treatments.

## 2. Methods

### 2.1. CMR-Based LV Model Construction

#### 2.1.1. CMR Imaging

The study consists of CMR images from seven cardiac amyloidosis patients before treatment (baseline) and at 6 or 9 months after the treatment (follow-up). The information of all patients is in Table 1. Each patient has been clinically classified as recovery, worsening, and stable (no obvious change). The assessment was based on the clonal response to chemotherapy and progression/regression on the extracellular volume. The biomechanical modeling analysis in this paper is blind to the clinical assessment and the CMR imaging acquisition, briefly summarized in Appendix A.1, has been described in detail elsewhere (Fontana et al., 2015).

**Table 1:** Cardiac amyloidosis patients and treatment details.

**Case**	**Age**	**Sex**	**Weight (kg)**	**Blood pressure (mmHg)**		**Gadolinium dosage (mL)**	
	**Baseline**		**Baseline**	**Baseline**	**Follow-up**	**Baseline**	**Follow-up**
1	62	M	110	79/42	96/61	22	17.8
2	55	F	78.9	106/71	131/80	15.8	16
3	54	F	75	115/68	108/71	16.2	15
4	70	M	67.8	94/62	96/61	13.6	13.1
5	65	M	57	111/69	107/71	11.4	12.3
6	59	F	87.8	112/69	129/83	17.6	21
7	72	M	75.4	106/66	108/71	15.1	11.5

#### 2.1.2. Ventricular Model Reconstruction

**Figure 1:**
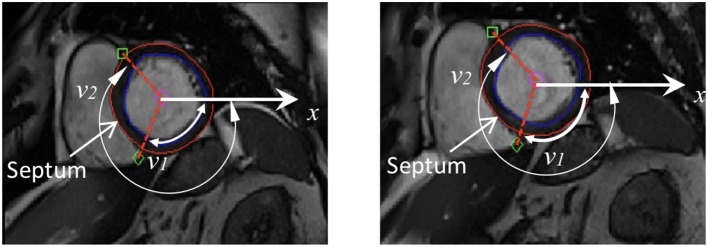
Definition of right ventricular insertion points at the basal plane in order to align LV geometries circumferentially at different time instances.

**Figure 2:**
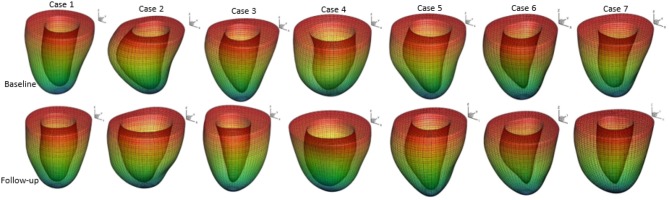
Generated LV geometrical models based on the CMR images and their meshes at early-diastole of seven patients in baseline and follow-up scans.

A prolate spherical coordinate system is used to reconstruct the LV geometry following the steps in Liu et al. (2009). Short-axis and long-axis cine images (Figure A1) at a total of 13 time instants in diastole are used to warp the LV geometry. The LV wall boundaries are manually segmented using an in-house Matlab code (Gao et al., 2017), and all short-axis LV wall boundaries are aligned to the images of the horizontal long axis, the vertical long axis, and the left ventricular outflow tract, respectively. In order to align the LV geometries along the long-axis at different times, we first determine the distance between the most-basal short-axis image and the mitral annulus ring, denoted as *d*_t_ at time *t*. Following this, the most-basal short-axis image is moved toward the annuls ring along the long axis with a distance of *d*_t_ − min(*d*_*i*_), (*i* = 1, …13, representing the *i*^*th*^ short axis image at diastole). Note that the long axis is defined by connecting the center of the LV base and the apex. In order to align the LV geometries circumferentially, the angles of right ventricular insertion points are defined in the basal plane (corresponding to the most-basal short-axis image), *v*_1_ near the inferior segment, and *v*_2_ near the anterior segment, as shown in Figure 1 at two different time instances. Then, for LV geometries at different times, the basal plane is aligned by matching the insertion points at *v*_1_, and the same number of elements are assigned to the septum circumferentially when generating the layered hexhedron mesh. Figure 2 shows the reconstructed LV at early-diastole, right after isovolumetric relaxation when the mitral valve just opens and the ventricular pressure is the lowest, for all patients both before and after treatment. This choice of reference configuration has been used in the literature (Genet et al., 2014; Gao et al., 2017).

**Figure 3:**
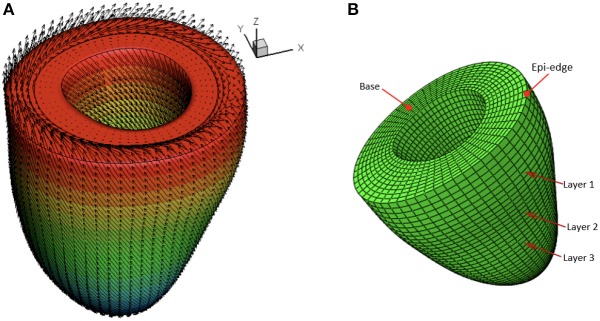
Schematic illustration of myofiber orientation **(A)** and selected three layers in the LV wall **(B)**.

A rule-based approach (Potse et al., 2006) is adopted to generate myofiber structure within the LV wall, with fibers varied linearly from −60^*o*^ at the epicardium to 60^*o*^ at the endocardium, and the sheet angle from −45^*o*^ at the epicardium to 45^*o*^ at the endocardium (see Figure 3A). Because the reconstructed LV geometries are fitted to one standard template mesh after long-axis and circumferential alignment, we may reasonably assume that elements with the same index from LV models at different times are co-registered. As shown in Figure 3B, three short-axis planes are further defined for extracting strains. Within each selected plane, 20 regions distributed equally along the circumferential direction are selected to obtain regional-average strains. In total, 60 circumferential strains are measured from CMR derived LV models.

Since each LV geometry has the same mesh connectivity, after co-registration of all LV geometries, a mapping can be established for every element of the LV geometry at different time frames. The deformation gradient related to the first time frame (at early-diastole) can be readily calculated as $𝔽=\frac{\partial \text{x}}{\partial \text{X}}$, where **X** and **x** are position vectors at the first and later time frames, respectively. We further obtain the circumferential direction (**c**) locally with respect to the long axis, and the transmural direction (**r**) pointing from endocardium to epicardium, and then the local longitudinal direction **l** = **r** × **c**, which follows local geometrical curvature (Gao et al., 2014a). This enables us to compute the circumferential and longitudinal strains as $E_{\text{cc}}=\text{c}\cdot \left(\frac{1}{2}\left({𝔽}^{T}𝔽-\text{I}\right)\text{c}\right)$ and $E_{\text{ll}}=\text{l}\cdot \left(\frac{1}{2}\left({𝔽}^{T}𝔽-\text{I}\right)\text{l}\right)$, in which **I** is the identity matrix. Note the reference state for strain calculation is early-diastole, not end-diastole which is usually adopted in clinics.

### 2.2. Personalized Biomechanical LV Model

#### 2.2.1. Constitutive Law

We use the Holzapfel-Ogden strain energy function to describe myocardial passive properties (Holzapfel and Ogden, 2009),

(1) $$\begin{array}{l} \text{Ψ}=\frac{a}{2b}\exp\left(b\left(I_{1}-3\right)\right)+\underset{i=\text{f},\text{s}}{\sum }\frac{a_{i}}{2b_{i}}\left[\exp\left(b_{i}{\left(\max\left(I_{4i},1\right)-1\right)}^{2}\right)-1\right]\\ \;+\frac{a_{\text{fs}}}{2b_{\text{fs}}}\left[\exp\left(b_{\text{fs}}I_{\text{8fs}}^{2}-1\right)\right] \end{array}$$

where *a*, *b*, *a*_f_, *b*_f_, *a*_s_, *b*_s_, *a*_fs_, *b*_fs_ are patient-dependent material parameters, and *I*_*j*_ (*j* = 1, 4f, 4s) are invariants of the right Cauchy-Green tensor. A more detailed description of the model (1) can be found in Holzapfel and Ogden (2009) and its applications in LV modeling should be referred to Göktepe et al. (2011), Wang et al. (2013), Gao et al. (2014b), and Wang et al. (2014). Differentiation of the strain energy function (1) with respect to the displacements and applying constraints related to various conservation laws leads to a set of equations that define the cardiac dynamics. These equations are solved numerically using finite element discretization, implemented in ABAQUS software 6.11.

#### 2.2.2. Boundary Conditions

Early-diastole is used as the reference configuration. The following boundary conditions are applied at the most basal plane (see Figure 3B),

(2) $$\begin{array}{l} \{\begin{array}{c} u_{x}^{\text{edge}}\left(t\right)=u_{x}^{\text{CMR}}\left(t\right),\;u_{y}^{\text{edge}}\left(t\right)=u_{y}^{\text{CMR}}\left(t\right),\\ u_{z}^{\text{edge}}\left(t\right)=0,\;\text{ on the epicardial edge}\\ u_{z}^{\text{base}}\left(t\right)=0,\;\text{ excluding the epicardial edge} \end{array} \end{array}$$

where $u_{x}^{\text{edge}}$, $u_{y}^{\text{edge}}$, $u_{x}^{\text{CMR}}$, $u_{y}^{\text{CMR}}$ are the displacements in the *x* and *y* directions determined from the model and the CMR images at the epicardial edge in the most basal plane (see Figure 3B).

The diastolic pressure profile is assumed to be linear between zero at early-diastole (*t* = 0 s) and *P*_ED_ at end-diastole (*t* = 1 s), following (Steendijk et al., 2004). *t* here is a pseudo simulation time. The values of *P*_ED_ should be patient-specific. However, as the pressure measurements are invasive, this information is not available from *in vivo* studies. On the other hand, the literature suggests that all amyloidosis patients have increased wall thickness and higher pressure compared with normal subjects (Kholova and Niessen, 2005; Quarta et al., 2012; Martinez-NaharrO et al., 2018). Hence, we assume *P*_ED_ is proportional to scaled LV wall volume as follows:

(3) $$\begin{array}{l} \frac{P_{\text{ED}}}{P_{\text{EDm}}}=\frac{V_{\text{wall}}/V_{\text{LV}}}{{\left(V_{\text{wall}}/V_{\text{LV}}\right)}^{m}}, \end{array}$$

where *P*_EDm_ is the mean end-diastolic pressure, taken to be 19 mmHg based on measurements in Plehn et al. (1992) and Boufidou et al. (2010). *V*_wall_/*V*_LV_ is the ratio of the LV wall volume to the LV chamber volume. Its mean value ${\left(V_{\text{wall}}/V_{\text{LV}}\right)}^{m}$ at early-diastole before treatment is 2.678. The scaled *P*_ED_ for each patient is listed in Table 2. This range of pressure values seems to be consistent with clinical observations of amyloidosis patients (Bhuiyan et al., 2011).

**Table 2:** Wall volume ratio and estimated end-diastolic pressure of the amyloidosis patients.

	***V_wall/vLV_***		*****P*****_**ED**_ **(mmHg)**	
**Case**	**Baseline**	**Follow-up**	**Baseline**	**Follow-up**
1	2.2066	2.4505	17.38	15.65
2	1.5379	1.8881	10.91	13.39
3	2.7021	3.8163	19.17	27.07
4	1.9746	1.6576	14.01	11.76
5	2.2003	2.6155	15.57	18.55
6	3.2463	3.196	23.03	22.67
7	3.5	5.0283	24.83	35.67

#### 2.2.3. Parameter Inference

For each amyloidosis patient, the material parameters in Equation (1) are inferred by using an optimization algorithm through minimizing an objective function (the difference between the model and imaged-derived P-V curve and circumferential strains). Sensitivity analysis in our previous study (Gao et al., 2015) shows the ranking order of the parameters, from the most significant to the least, is

(4) $$\begin{array}{l} a>a_{\text{fs}}>a_{\text{f}}>b_{\text{f}}>b\gg b_{\text{fs}}>a_{\text{s}}\approx b_{\text{s}}. \end{array}$$

This allows us to divide the parameters in Equation (1) into two groups, the first group includes *a*, *b*, *a*_f_, *b*_f_, *a*_fs_, and the second group involves *a*_s_, *b*_s_, *b*_fs_. The first group may be determined with higher accuracy than the second group because of the higher sensitivity to clinical measurements, although this may not be true all the time as the material model is strongly nonlinear. As such, a two-step approach is used. In the first step, parameters of the first group are determined by minimizing the following objective function with the parameters in the second group taking the values from Gao et al. (2015) for healthy volunteers estimated at *P*_EDm_ = 8 mmHg (*a*_s_ = 0.5426 kPa, *b*_s_ = 1.5998, *b*_fs_ = 3.3900):

(5) $$\begin{array}{l} \{\begin{array}{c} F_{\text{VE}}=F_{V}+F_{E}\\ F_{V}=w_{v}\overset{n_{\text{time}}}{\underset{i=1}{\sum }}{\left[\left(V_{i}^{\text{FEA}}-V_{i}^{\text{CMR}}\right)/V_{i}^{\text{CMR}}\right]}^{2}\\ F_{E}=\frac{w_{E}}{n_{\text{layer}}n_{\text{reg}}}\overset{n_{\text{layer}}}{\underset{k=1}{\sum }}\overset{n_{\text{reg}}}{\underset{j=1}{\sum }}\overset{n_{\text{time}}}{\underset{i=1}{\sum }}{\left(\bar{E}{\text{cc}}_{i,j,k}^{\text{FEA}}-\bar{E}{\text{cc}}_{i,j,k}^{\text{CMR}}\right)}^{2} \end{array} \end{array}$$

where *n*_layer_ is the number of layers considered, *n*_layer_ = 3 indexed by *k*; *n*_time_ is the number of time steps, *n*_time_ = 13 indexed by *j*; *n*_reg_ is the number of regions in each layer considered, *n*_reg_ = 20 indexed by *i*; $V_{i}^{\text{FEA}}$, $V_{i}^{\text{CMR}}$ are the LV chamber volumes from the FEA and CMR images, respectively; $Ē{\text{cc}}_{i,j,k}^{\text{FEA}}$, $Ē{\text{cc}}_{i,j,k}^{\text{CMR}}$ are the mean circumferential strains in 20 regions from the FEA and CMR images, respectively; *w*_*v*_, *w*_*E*_ are the weights. Note we do not include the longitudinal strains in our objective function because the uncertainty of estimating longitudinal strains is much larger than the circumferential strains. This is because the simplified long-axial alignment we use does not account for the out-of-plane motion along the long axis. We are unable to quantify the out-of-plane motion due to the lack of necessary features in cine images. 3D strain imaging such as tagging allows one to include longitudinal strains (Nikou et al., 2016) or the displacement fields (Asner et al., 2016) in the objective function, but it is not used routinely in clinics. It is for this reason many published studies using *in vivo* cine images mainly used measured volume (Genet et al., 2014; Palit et al., 2018), or regional circumferential strains along with measured volume in the objective function (Gao et al., 2015).

After obtaining the optimal five constants in the first group, we proceed to infer all parameters but found *a*_s_, *b*_s_, and *b*_fs_ are insensitive to the optimization. Therefore we first focus on the ratios of parameters: *a*_s_/*a*_fs_, *a*_f_/*a*_s_, *b*_s_/*b*_fs_, *b*_f_/*b*_s_. A range of values for these ratios can be found from the literature (Gao et al., 2015; Palit et al., 2018) from which we derive the linear regressions,

(6) $$\begin{array}{l} \{\begin{array}{c} a_{\text{s}}/a_{\text{fs}}=1.72a_{\text{f}}/a_{\text{s}}-3.65\\ b_{\text{s}}/b_{\text{fs}}=-0.43b_{\text{f}}/b_{\text{s}}+1.61 \end{array}. \end{array}$$

The first equation of (6) shows that *a*_s_ is the only unknown after the first optimization step,

(7) $$\begin{array}{l} a_{\text{s}}=\frac{1}{2}[1.72a_{\text{fs}}+\sqrt{{\left(3.65a_{\text{fs}}\right)}^{2}+4\times 1.72a_{\text{f}}a_{\text{fs}}}]. \end{array}$$

In the second equation of (6), two unknowns remain, *b*_s_ *b*_fs_. Let ξ = *b*_f_/*b*_fs_, we write

(8) $$\begin{array}{l} \{\begin{array}{l} b_{s}=b_{\text{f}}/\xi \\ b_{\text{fs}}=\frac{b_{\text{f}}}{\xi (1.61-0.43\xi )} \end{array} \end{array}$$

It is easy to see that ξ ∈ [1.44, 2.96] from data in Gao et al. (2015) and Palit et al. (2018). Thus in the second step, ξ is optimized by minimizing the objective function *F*_*E*_ in Equation (5).

The flowchart of the two-step optimization method and the inferred parameters are given in Appendix A.2. The uncertainties of these parameters are evaluated using the residual bootstrap method (Efron and Tibshirani, 1986), as described in Appendix A.2.

### 2.3. Shape Analysis and Statistical Classification

**Figure 4:**
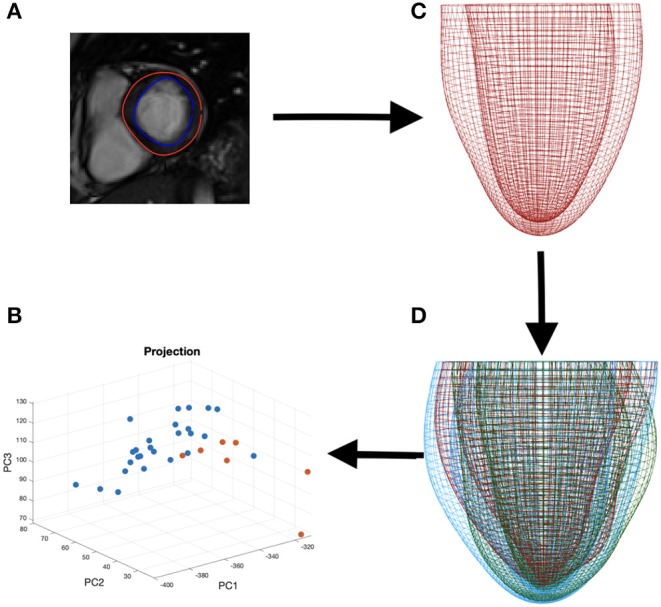
The process of data segmentation and dimensionality reduction. First we segment the outer and inner walls of the LV **(A)** allowing us to construct a mesh representation of the LV **(C)**. We then extract the main variations in a sample of LVs **(D)** allowing us to represent the geometries in a lower dimensional space **(B)**.

For feature comparison between the amyloidosis patients and control, we make use of the LV geometries of 26 healthy subjects from our previous study (Gao et al., 2017). Basic characteristics for the healthy volunteers are: ages: 45±15, sex (male: female): 15 : 11, systolic blood pressure (mmHg): 145.6 ± 31.4, diastolic blood pressure (mmHg): 83 ± 15, LV EF(%):57 ± 5, LV end-diastolic-volume (mL): 126 ± 21, LV end-systolic-volume (mL): 55±14. Geometry reconstruction follows the same procedure as for the amyloidosis patients (see section 2.1.2). All the LV geometries are fitted to one template LV mesh, with 5,792 vertices from the endocardial and epicardial surfaces, extracted from CMR images with the same imaging orientation as shown in Figure 4 (i.e., the chest wall in the left side of the short-axis images). Note the vertices inside the ventricular wall are excluded, and each vertex has three coordinate components. Thus, a Cartesian coordinate representation lies in the 17,376 dimensional space, necessitating the use of dimensionality reduction techniques for consideration of the geometry in the context of classification and data visualization (see Figure 4).

To analyse the LV shape change, we first need to represent the LV in a low dimensional space. Principal component analysis (PCA) relies on, successively, finding the principal directions of variation in the data where the amount of variation explained by the eigenvectors (principal components) can be quantified using the corresponding eigenvalues (Bishop, 2006). If we begin with data in *n* dimensions, then projecting onto the first *m* < *n* principal components provides us with a lower dimensional representation of the data while preserving variations in the data captured by these first *m* principal components. By construction, PCA assumes a linear mapping into the lower dimensional space and constrains the principal directions of variation to be orthogonal to one another (Shlens, 2014). Similarly, there is an implicit assumption of Gaussianity since we assume that dependence between data points is fully specified by the first two moments (mean and variance). These limitations of PCA can be overcome by considering a more flexible, non-linear, dimensionality reduction technique. Methods such as an autoencoding neural network could be considered however, for sparse data sets, as available in our study, parameter tuning will either lead to overfitting, or (if properly regularized) lose the non-linear model flexibility that motivated the method in the first place. For visualization purposes, t-Distributed Stochastic Neighbor Embedding (t-SNE) (van der Maaten and Hinton, 2008) provides a non-linear projection of the data into a lower dimensional space by minimizing a KL divergence between conditional probabilities of nearest neighbors in both spaces. However, since an explicit transformation is never learned, the method is limited to only data visualization. More details of dimensional reduction is given in Appendix A.3.

For the purpose of classification, we can first consider a supervised method similar to PCA, namely linear discriminant analysis (LDA) (Bishop, 2006). Whereas PCA finds the direction of maximal variation without taking classes into account, LDA attempts to find a lower dimensional projection for separation of the two classes (in this case, healthy volunteers and amyloidosis patients). LDA is restricted by assumptions of linearity and Gaussianity. To overcome these constraints, we will also consider a kernel support vector machine (SVM), which allows for the possibility of non-linear boundaries between the groups in the dataset by the introduction of a kernel function (Murphy, 2012).

## 3. Results

### 3.1. Analysis of Mechanical Features

#### 3.1.1. Material Parameters

The optimization procedure is given in Appendix A.2, followed by uncertainty quantification using the bootstrap method. The inferred material parameters for the seven patients and the corresponding errors are listed in **Table 5**, and Tables A2, A3. However, it is not easy to see the pattern of parameter changes directly given the potential correlations between these parameters, and the lack of uniqueness. To assess the mechanical features, below we analyze the mechanical response of the cardiac amyloidosis patients during the disease progression using our FE models with the inferred parameters.

#### 3.1.2. p-V Curve

**Figure 5:**
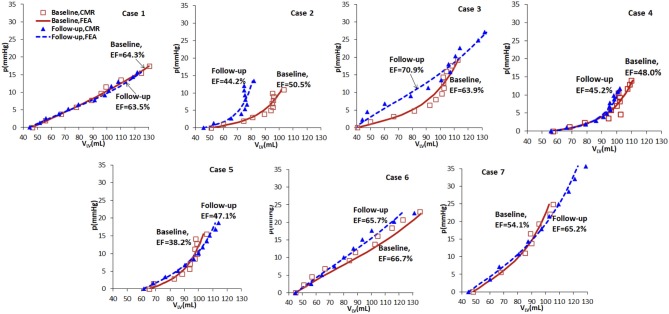
p-V curves of the seven cases in baseline (red) and follow-up (blue), from CMR images (symbols) and FE models (lines).

Based on the wall-thickness scaled *P*_ED_, the p-V curves estimated from the FE models are shown in Figure 5, which are compared with the corresponding results when the volume is estimated directly from CMR images. Figure 5 shows that there are little changes in the p-V curves of Cases 1, 4, 5, 7 from baseline to follow-up, but dramatic changes for Cases 2, 3, 6. The end-diastolic volumes of Cases 3, 5, and 7 increased compared to the baseline values, suggesting a ventricular dilation, while the end-diastolic volumes of Cases 1, 2, 4, and 6 decreased, especially for Case 2.

#### 3.1.3. Stress-Stretch Response

**Figure 6:**
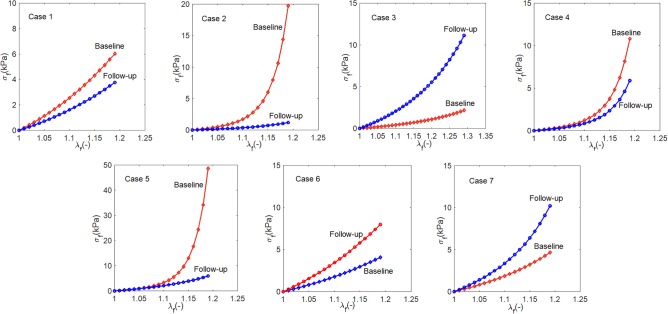
The stress-stretch responses of the patients at the baseline (red) and follow-up (blue).

The myocardium stress-stretch response along the myofiber direction for each patient can now be obtained from a pseudo uni-axial test of the myocardium using the material parameters estimated with perfectly aligned myofibers in one direction. The results are plotted in Figure 6. The stress-stretch curves of Cases 1, 2, 4, 5, and 6 in the follow-up are less stiff than in the baseline, in contrast to Cases 3 and 7. Note that in Case 6, the difference in the baseline and follow-up is very small.

#### 3.1.4. 3D Strain and Stress Distribution

**Figure 7:**
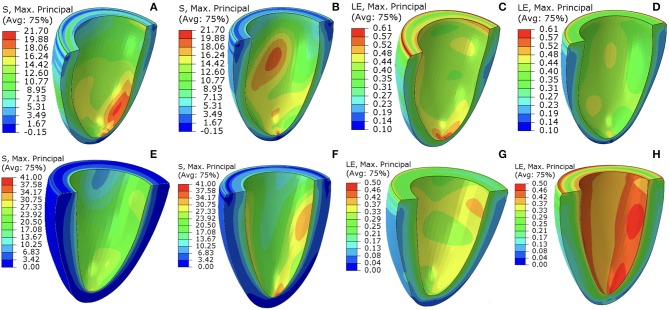
The first principal stress contours at baseline **(A,E)** and follow-up **(B,F)**. The corresponding (logarithmic) strain contours are shown in **(C,D,G,H)**. **(A–D)** are for Case 1, **(E–H)** are for Case 7. The unit of the stresses is kPa.

Figure 7 shows the first principal strain and stress of two different patients (Case 1 and Case 7) at baseline and follow-up. Clearly, the progression is very different in these two subjects. In Case 1, the first principal strain is slightly lower in the follow-up (Figure 7B) compared to the baseline (Figure 7A), particularly in the apical region. The strain patterns are somewhat different, but the maximum strain does not increase in the follow-up (Figure 7C vs. Figure 7D). However, for Case 7, there is a dramatic increase in the first principal stress level in the follow-up (Figure 7E) from the baseline Figure 7F. The strain patterns are also very different. In terms of the LV shape, not much change is seen in Case 1, but significant axial elongation is observed in Case 7.

### 3.2. Shape Analysis

PCA permits an intuitive representation of the variations in the data via the modes of variation (See Appendix A.3, top of Figure A3), obtained by perturbing the mean LV shape along each of the principal components. For our dataset, mode 1 is clearly related to overall size of the LV, mode 2 appears to represent thickness of the LV wall and mode 3 is related to the horizontal shape. Considering the absence of group indicators in this method, it is interesting to observe the second mode of variation, showing that within the dataset one of the largest sources of LV variation appears to be wall thickness. In the central plot on the bottom of Figure A3 we see the projection of the data onto this second principal component where, as expected, there is some separation of the amyloidosis patients from the healthy volunteers once we isolate this source of variation. This separation can also be observed by considering the median distance (wall thickness) between points on the epicardium and endocardium of healthy volunteers and amyloidosis patients, found to be 0.77 and 1.11 cm for healthy volunteers and amyloidosis patients respectively. Letting wall thickness be taken as the median distance between the epicardium and endocardium walls, we obtain a *p* < 0.05 for a test of a difference between median wall thickness of healthy volunteers and amyloidosis patients using Mood's median test (these results are consistent with a *t*-test and a rank sum test).

Before studying amyloidosis progression prediction, we perform classification of the seven amyloidosis patients at baseline compared with a set of 26 healthy volunteers based on geometries alone, assessing the performance of LDA and a kernel SVM. Sensitivity (the ratio of the correctly predicted positive observations to all observations in the positive class) and specificity (the ratio of correctly predicted negative observations to all observations in the negative class) scores for these methods, obtained using a leave-one-out cross validation (LOOCV) procedure, are given in Table 3 along with an overall accuracy quantification obtained using the F_1_ score:

(9) $$\begin{array}{l} F_{1}=2\frac{TP}{2TP+FP+FN} \end{array}$$

where *TP* is number of true positives, *FP* is number of false positives and *FN* is number of false negatives. The F1 score is the harmonic mean of the precision (the ratio of correctly predicted positive observations to the total predicted positive observations) and the recall (also called sensitivity). We use the harmonic rather than the arithmetic mean to penalize the improvement of one of the scores at the expense of the other.

Accuracy of both the linear and non-linear methods is encouraging, suggesting the discriminative properties of the LV geometry with regards to amyloidosis.

**Table 3:** Sensitivity, specificity, and F1 scores for classifying geometries using LDA and Kernel SVM.

**Method**	**LDA**	**Kernel SVM**
Specificity	0.92	1
Sensitivity	0.71	0.86
*F*_1_	0.71	0.92

### 3.3. Classification and Prediction

#### 3.3.1. Selected Markers for Classification

The analysis above shows that no single marker provides a clear indication for disease progression. A combination of multiple features must be considered. To this end, we summarize the representative markers below that may contribute to the growth and remodeling of myocardium.

1. *V*_wall_/*V*_LV_, which reflects the wall thickness change relative to the ventricular volume. A lower value of *V*_wall_/*V*_LV_ means the wall becomes thinner (recovery);
2. Ē_cc_, the circumferential strain at end-diastole averaged from the three planes. A larger value of Ē_cc_ indicates that the LV is more compliant in the circumferential direction (recovery);
3. Ē_ll_, the longitudinal strain at end-diastole averaged from the three planes. A larger value of Ē_ll_ indicates that the LV is more compliant in the longitudinal direction (recovery);
4. ${\bar{\sigma }}_{1}$, the principal stress at end-diastole averaged from the three planes. A lower value of ${\bar{\sigma }}_{1}$ means the tissue is less stressed (recovery);
5. *W*, work done by pressure during diastolic filling, because it is not straightforward to compare different p-V curves, we compare the area-under-the-curve, which is defined as
  $$\begin{array}{l} W=\int_{\text{0}}^{\text{diastole}}pdV_{\text{LV}}. \end{array}$$
  A higher value of *W* in the follow-up means more work is required to maintain the heart function, corresponding to worsening.
6. The average slope $\bar{f}$ of the stress-stretch curve σ − λ. A steeper σ − λ in the follow-up indicates the myocardium becomes stiffer (worsening).
7. Shape features, obvious shape changes compared to control indicate worsening.

The markers used for follow-up cardiac amyloidosis status prediction are divided into three classes: (1) geometrical markers (features 1–3), (2) biomechanical markers (features 4–6), and (3) LV shape markers (feature 7). Geometrical markers include normalized LV wall thickness, which has been applied clinically. Biomechanical markers are discussed in section 3.2.

#### 3.3.2. Shape Classification and Prediction

**Figure 8:**
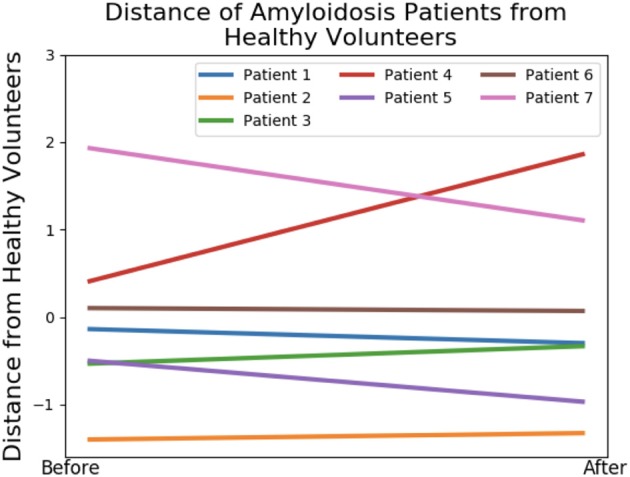
Shape analysis of the amyloidosis patients. This plot was produced using LDA during an initial analysis of the data, before any patient recovery labels were known. The y-axis provides a measure of distance from the group of healthy volunteers and the x-axis provides two timepoints: before and after treatment.

We first study the amyloidosis patient recovery using shape features. As we do not know what is a “healthier” shape for LV, we make use of the control data from our previous study (Gao et al., 2017). The analysis is conducted by projecting the seven patients onto LDA components and measuring distances from the group of healthy volunteers before and after treatment. Pre-processing with PCA is necessary, removing collinearity and preventing singularities in the LDA calculations (this is the result of all meshes being formed by the same base LV mesh). Figure 8 presents these distances where a negative gradient is a sign of movement toward healthy volunteers. Only patients 1, 5, 6, and 7 appear to improve as a result of the treatment. This analysis is performed using leave-one-out-cross-validation where in each case one amyloidosis patient is left out of the training set. These movements toward or away from healthy volunteers provide the shape marker in Table 4 where values of the six markers in the previous section are also provided. Further details on computation of the first six markers are provided in Appendix A.2.

**Table 4:** Classification for the amyloidosis patients based on various markers.

**Marker**	***V****_wall_/V*_LV_	****Ē_cc_****	****Ē**_ll_**	**W**	**${\bar{\sigma }}_{1}$**	**$\bar{f}$**	**Shape**
**Case**	**(Follow-up** **−** **Baseline)/Baseline × 100%**						
1	11.05	10.66 (0.06)	−10.72 (0.092)	−18.86 (0.05)	−18.20 (0.11)	−33.86 (0.12)	down
2	22.77	25.24 (0.10)	−51.86 (0.12)	−13.51 (0.03)	−38.59 (0.18)	−92.69 (0.09)	up
3	41.2	−4.77 (0.03)	65.03 (0.16)	168.22 (0.06)	39.79 (0.31)	428.19 (2.26)	up
4	−16.05	2.65 (0.10)	−10.63 (0.11)	−30.70 (0.11)	−6.53 (0.07)	−40.92 (0.69)	up
5	18.87	70.20 (0.18)	7.26 (0.18)	108.82 (0.09)	−8.50 (0.07)	−93.09 (0.08)	down
6	−1.55	−29.86 (0.03)	0.74 (0.06)	−14.37 (0.04)	13.36 (0.03)	193.59 (1.52)	down
7	43.67	9.33 (0.08)	83.90 (0.14)	154.70 (0.15)	42.12 (0.17)	168.10 (2.45)	down
Better:	4,6	1,2,4,5,7	3,5,6,7	1,2,4,6	1,2,4,5	1,2,4,5	1,5,6,7
Worse:	1,2,3,5,7	3,6	1,2,4	3,5,7	3,6,7	3,6,7	2,3,4

*The criterion for improvement for the first 6 markers is based on physiology, as described in section 3.3.1. The criterion for improvement for “shape” (last column) is based on the statistical analysis described in section 3.3.2. The uncertainty intervals for the biomechanical markers (columns 5–7) are obtained from the residual bootstrap analysis described in Appendix A.2, A.4. Note the sign of the values does not change when taking this uncertainty into account*.

Now that we have quantified the shape features, we can summarize the changes of all the markers from our model in Table 4. The original values of these markers at the baseline and follow up as well as the uncertainty quantification are provided in Appendix A.4.

## 4. Discussion

Using a modeling approach, we have studied the predictive power of the mechanical and geometric markers with respect to amyloidosis classification. Of great interest is the relation of amyloidosis progression with these markers, and which ones have greater predictive power. We find that, due to the complexity of the LV disease, no single marker can provide the whole picture of the disease progression. Indeed, as shown in Table 4, some markers give opposite predictions for the same case. To overcome this issue, we made use of the recovery score for each patient based on the predictions of all the markers studied.

Table 5 summarizes the results of predicting recovery of amyloidosis patients. The recovery score refers to a classification done based on Table 4, which was found before the patient labels were made available. The recovery score is obtained as the proportion of “better” predictions in Table 4. In other words, we take the number of recovery scores and divide by the total so if a patient is said to recover by 3 out of 7 markers, then the recovery score is 3/7. The small sample size here severely limits significance of these results, but by consulting a committee of weak classifiers we seek to obtain more conclusive results. All patients were diagnosed with heart failure and all of them had NYHA class 2 at presentation. However, some cases (e.g., Case 1) became class 1 after treatment, and others (e.g., case number 7) became class 3 on the second follow up. Hence, the clinical assessments can be made. This is used to compare to our recovery score in Table 4, with a good overall agreement, particularly in cases (1, 3, 4, 6, 7). Notice that although we have computed the recovery scores, we do not know the corresponding range of recovery scores to the clinical statuses (of recovery, stable or worsening). If we declare all scores above 0.5 correspond to stable or recovery, then 6 out of 7 predictions are accurate. Case 2 is predicted wrong, but the score is almost at the boundary.

**Table 5:** Model predication vs. clinical assessment.

**Patient**	**1**	**2**	**3**	**4**	**5**	**6**	**7**
Recovery score	0.71	0.57	0.14	0.57	0.71	0.71	0.43
Clinical assessment	Recovery	Worsening	Worsening	Stable	Stable	Recovery	Worsening

*The higher the score, the more likely is the recovery, and vice versa*.

Despite the encouraging results from the double blind test shown in Table 5, limitations of our work must be discussed. This is a proof of concept study in the goal of classifying disease progression after treatment in amyloidosis patients. Thus, although the concept of the approach is deemed to be rather promising, it is important to exercise caution when interpreting the statistical results presented in this paper, as the lack of data reduces significance of the statistical analysis, as well as the dimensionality reduction results.

There are two issues that can impact the stress values we estimate. The first is that it is known that the eight parameters in the HO model are coupled and not independent. Therefore, each parameter may not be uniquely determined. However, it is not the individual change of the parameters that we look for, but the collective effects of all the parameters. For example, it has been shown that the stress-strain curves can be more robustly estimated despite the inter-correlations of the parameters, as shown in our previous study (Gao et al., 2015), for different measurement noise levels or initial values. To quantify the parameter uncertainty in our paper, we have carried out a residual bootstrap analysis (Efron and Tibshirani, 1986). Our uncertainty quantification follows a three-tier approach. At the bottom tier, we apply the residual bootstrap analysis to estimate the estimation uncertainty of the biomechanical parameters, which are defined below Equation (1). The methodological details are described in Appendix A.2. Note that the bootstrap analysis takes two effects into consideration: intrinsic uncertainty as a consequence of measurement noise, and algorithmic uncertainty as a consequence of potential convergence of the optimization algorithm to local optima of the objective function. At the middle tier, we use the bootstrap distributions of the biomechanical parameter estimates from the bottom tier to obtain the corresponding distributions of the biomechanical markers, which were introduced in section 3.3.1. The results can be found in Appendix A.4. At the highest tier, we use the uncertainty of the biomechanical markers to determine the uncertainty of the recovery scores. The methodological details can be found in Appendix A.4, and the results are in Table 4.

The second issue is the significant assumption we made on the end-of-diastole pressure for the patients since invasive pressure measurements are not available. We assumed that there is a proportional relationship between the pressure and wall volume, inspired by data from the literature. This assumption increased the uncertainty of the final stress values we computed. However, we would like to state that it is not the absolute stress values, but the relative change (follow up vs. acute), that matters in our evaluations. Clearly, Table 4 shows that the recovery scores are not affected by the uncertainty intervals since, within the interval provided by the uncertainty propagation outlined in Appendix A.4, the recovery indicator does not change. We also estimated the recovery scores based on markers from the image-based strain and shape analysis alone, and found that the prediction is not as good, in that two cases (1 and 7) are predicted wrong if we exclude the stresses related markers. However, we noted that some of the individual scores give nearly opposite results. For example, in Table 4, two strain and shape indicators show that case 7 is getting better, but the three stress and wall thickness indicators show it is getting worse. Hence, not all indicators give a positive contribution to the overall score. This highlights the complexity of the pathological system, and indicates that no single biomarker studied is able to predict the amyloidosis progression. We tentatively suggest that competing mechanisms may be in play during patients' recovery. For instance, the increased strains (showing recovering) in Case 7 are accompanied by the increased stresses (showing worsening). This may imply that the more stiffened myocardium over-weights the benefits of the smaller strains. Therefore, it seems that multiple markers are required to give a balanced view for the overall picture. We remark again that our observations need to be supported by a larger sample size, as with a small sample size it is difficult to distinguish systematic effects from random fluctuations.

Other modeling limitations should also be mentioned. In this paper, amyloidosis LV is regarded as homogeneous material. The loaded early diastolic configuration is used as the reference configuration which excludes the effect of residual stresses. Our alignment of LV geometries at different times is based on a simplified linear registration approach. Nonlinear methods, such as deformable registration approaches (Rueckert et al., 1999), the large deformation deformetric metric mapping (Durrleman et al., 2014), may provide more accurate geometry co-registrations. These issues need to be addressed in future work.

## 5. Conclusion

A proof of concept analysis of cardiac amyloidosis progression has been obtained by projecting a group of amyloidosis patients onto linear discriminant analysis components and measuring distances from the group of healthy volunteers before and after treatment. Extensive mechanical, geometrical, and shape markers are included in the analysis for the first time for cardiac amyloidosis patients. A promising agreement with clinical observation is achieved in predicting disease progression following medical treatments in a double blind test. Although these results should be interpreted with caution due to a small sample size, the methodology of using statistical analysis and multiple markers, in particular the shape analysis, can play a powerful role in clinical translation in the future when used in large samples with new and automatic image segmentation methods.

## Data Availability Statement

All datasets generated for this study are included in the article/Supplementary Material.

## Ethics Statement

Ethical approval was obtained from the Joint University College London/University College London Hospitals Research Ethics Committee (REC reference: 07/H0715/101). All research-related procedures were performed in accordance with local guidelines and regulations. The patients/participants provided their written informed consent to participate in this study.

## Author Contributions

XL, HG, and DH designed the study. WL and AL developed the mechanical and statistical models. AM-N, MF, and PH acquired the CMR scans. SB, RJ, and JC initiated the research idea and helped with image analysis. CB provided clinical insight. All participated in writing the manuscript.

## Conflict of Interest

RJ and JC were employed by the company GlaxoSmithKline, plc, UK. The University of Glasgow holds research and/or consultancy agreements with Siemens Healthcare and GlaxoSmithKline plc for work done by XL, CB, and others.

## References

[B1] Asner L. Hadjicharalambous M. Chabiniok R. Peresutti D. Sammut E. Wong J.. (2016). Estimation of passive and active properties in the human heart using 3D tagged MRI. Biomech. Model. Mechanobiol. 15, 1121–1139. 10.1007/s10237-015-0748-z 26611908 PMC5021775

[B2] Bhuiyan T. Helmke S. Patel A. R. Ruberg F. L. Packman J. Cheung K.. (2011). Pressure-volume relationships in patients with transthyretin (ATTR) cardiac amyloidosis secondary to V122I mutations and wild-type transthyretin: Transthyretin cardiac amyloid study (TRACS). Circulation 4, 121–128. 10.1161/CIRCHEARTFAILURE.109.910455 21191093 PMC4939845

[B3] Bishop C. M. (2006). Pattern Recognition and Machine Learning (Information Science and Statistics). Berlin; Heidelberg: Springer-Verlag.

[B4] Boufidou A. Mantziari L. Paraskevaidis S. Karvounis H. Nenopoulou E. Manthou M.-E.. (2010). An interesting case of cardiac amyloidosis initially diagnosed as hypertrophic cardiomyopathy. Hellenic J. Cardiol. 51, 552–557. Available online at: https://www.hellenicjcardiol.org/archive/full_text/2010/6/2010_6_552.pdf 21169191

[B5] Buss S. J. Emami M. Mereles D. Korosoglou G. Kristen A. V. Voss A.. (2012). Longitudinal left ventricular function for prediction of survival in systemic light-chain amyloidosis: incremental value compared with clinical and biochemical markers. J. Am. Coll. Cardiol. 60, 1067–1076. 10.1016/j.jacc.2012.04.043 22883634

[B6] Carroll J. D. Gaasch W. H. McAdam K. P. (1982). Amyloid cardiomyopathy: characterization by a distinctive voltage/mass relation. Am. J. Cardiol. 49, 9–13. 10.1016/0002-9149(82)90270-3 6459025

[B7] Chapelle D. Felder A. Chabiniok R. Guellich A. Deux J.-F. Damy T. (2015). Patient-specific biomechanical modeling of cardiac amyloidosis–a case study, in International Conference on Functional Imaging and Modeling of the Heart (Maastricht: Springer), 295–303. 10.1007/978-3-319-20309-6_34

[B8] Chew C. Ziady G. M. Raphael M. J. Oakley C. M. (1975). The functional defect in amyloid heart disease: the stiff heart syndrome. Am. J. Cardiol. 36, 438–444. 10.1016/0002-9149(75)90891-7 1190048

[B9] Dungu J. N. Valencia O. Pinney J. H. Gibbs S. D. Rowczenio D. Gilbertson J. A.. (2014). CMR-based differentiation of AL and ATTR cardiac amyloidosis. JACC 7, 133–142. 10.1016/j.jcmg.2013.08.015 24412186

[B10] Durrleman S. Prastawa M. Charon N. Korenberg J. R. Joshi S. Gerig G.. (2014). Morphometry of anatomical shape complexes with dense deformations and sparse parameters. NeuroImage 101, 35–49. 10.1016/j.neuroimage.2014.06.043 24973601 PMC4871626

[B11] Efron B. Tibshirani R. (1986). Bootstrap methods for standard errors, confidence intervals, and other measures of statistical accuracy. Stat. Sci. 1, 54–75. 10.1214/ss/1177013815

[B12] Fontana M. Pica S. Reant P. Abdel-Gadir A. Treibel T. A. Banypersad S. M.. (2015). Prognostic value of late gadolinium enhancement cardiovascular magnetic resonance in cardiac amyloidosis. Circulation 132, 1570–1579. 10.1161/CIRCULATIONAHA.115.016567 26362631 PMC4606985

[B13] Frenzel H. Schwartzkopff B. Kuhn H. Lösse B. Thormann J. Hort W.. (1986). Cardiac amyloid deposits in endomyocardial biopsies: light microscopic, ultrastructural, and immunohistochemical studies. Am. J. Clin. Pathol. 85, 674–680. 10.1093/ajcp/85.6.674 3518402

[B14] Gao H. Aderhold A. Mangion K. Luo X. Husmeier D. Berry C. (2017). Changes and classification in myocardial contractile function in the left ventricle following acute myocardial infarction. J. R. Soc. Interface 14:20170203. 10.1098/rsif.2017.0203 28747397 PMC5550971

[B15] Gao H. Carrick D. Berry C. Griffith B. E. Luo X. (2014a). Dynamic finite-strain modelling of the human left ventricle in health and disease using an immersed boundary-finite element method. IMA J. Appl. Math. 79, 978–1010. 10.1093/imamat/hxu029 27041786 PMC4816497

[B16] Gao H. Li W. Cai L. Berry C. Luo X. (2015). Parameter estimation in a holzapfel–ogden law for healthy myocardium. J. Eng. Math. 95, 231–248. 10.1007/s10665-014-9740-3 26663931 PMC4662962

[B17] Gao H. Wang H. Berry C. Luo X. Griffith B. E. (2014b). Quasi-static image-based immersed boundary-finite element model of left ventricle under diastolic loading. Int. J. Numer. Methods Biomed. Eng. 30, 1199–1222. 10.1002/cnm.2652 24799090 PMC4233956

[B18] Genet M. Lee L. C. Nguyen R. Haraldsson H. Acevedo-Bolton G. Zhang Z.. (2014). Distribution of normal human left ventricular myofiber stress at end diastole and end systole: a target for *in silico* design of heart failure treatments. J. Appl. Physiol. 117, 142–152. 10.1152/japplphysiol.00255.2014 24876359 PMC4101610

[B19] Gertz M. Dispenzieri A. Grogan M. Kumar S. Leung N. Maurer M. (2013). Amyloidosis Awareness. Amyloidosis Support Groups.

[B20] Göktepe S. Acharya S. Wong J. Kuhl E. (2011). Computational modeling of passive myocardium. Int. J. Numer. Methods Biomed. Eng. 27, 1–12. 10.1002/cnm.1402 PMC4567385 23798328

[B21] Grogan M. Dispenzieri A. Gertz M. A. (2017). Light-chain cardiac amyloidosis: strategies to promote early diagnosis and cardiac response. Heart 103, 1065–1072. 10.1136/heartjnl-2016-310704 28456755 PMC5566095

[B22] Holzapfel G. A. Ogden R. W. (2009). Constitutive modelling of passive myocardium: a structurally based framework for material characterization. Philos. Trans. R. Soc. Lond. A Math. Phys. Eng. Sci. 367, 3445–3475. 10.1098/rsta.2009.0091 19657007

[B23] Kholova I. Niessen H. (2005). Amyloid in the cardiovascular system: a review. J. Clin. Pathol. 58, 125–133. 10.1136/jcp.2004.017293 15677530 PMC1770576

[B24] Koyama J. Ray-Sequin P. A. Falk R. H. (2003). Longitudinal myocardial function assessed by tissue velocity, strain, and strain rate tissue doppler echocardiography in patients with al (primary) cardiac amyloidosis. Circulation 107, 2446–2452. 10.1161/01.CIR.0000068313.67758.4F 12743000

[B25] Liu D. Hu K. Niemann M. Herrmann S. Cikes M. Störk S.. (2013). Impact of regional left ventricular function on outcome for patients with al amyloidosis. PLoS ONE 8:e56923. 10.1371/journal.pone.0056923 23520459 PMC3592864

[B26] Liu Y. Wen H. Gorman R. C. Pilla J. J. Gorman J. H. III. Buckberg G.. (2009). Reconstruction of myocardial tissue motion and strain fields from displacement-encoded MR imaging. Am. J. Physiol. Heart Circul. Physiol. 297, H1151–H1162. 10.1152/ajpheart.00074.2009 19561315 PMC2755977

[B27] Martinez-NaharrO A. Hawkins P. N. Fontana M. (2018). Cardiac amyloidosis. Clin. Med. 18, s30–s35. 10.7861/clinmedicine.18-2-s30 29700090 PMC6334035

[B28] Murphy K. P. (2012). Machine Learning: A Probabilistic Perspective. The MIT Press.

[B29] Nikou A. Dorsey S. M. McGarvey J. R. Gorman J. H. Burdick J. A. Pilla J. J.. (2016). Computational modeling of healthy myocardium in diastole. Ann. Biomed. Eng. 44, 980–992. 10.1007/s10439-015-1403-7 26215308 PMC4731326

[B30] Nucifora G. Muser D. Morocutti G. Piccoli G. Zanuttini D. Gianfagna P.. (2014). Disease-specific differences of left ventricular rotational mechanics between cardiac amyloidosis and hypertrophic cardiomyopathy. Am. J. Physiol. Heart Circul. Physiol. 307, H680–H688. 10.1152/ajpheart.00251.2014 24993044

[B31] Palit A. Bhudia S. K. Arvanitis T. N. Turley G. A. Williams M. A. (2018). *In vivo* estimation of passive biomechanical properties of human myocardium. Med. Biol. Eng. Comput. 56, 1615–16317. 10.1007/s11517-017-1768-x 29479659 PMC6096751

[B32] Petre R. E. Quaile M. P. Wendt K. Houser S. R. Wald J. Goldman B. I.. (2005). Regionally heterogeneous tissue mechanics in cardiac amyloidosis. Amyloid 12, 246–250. 10.1080/13506120500386824 16399650

[B33] Plehn J. F. Southworth J. Cornwell III G. G. (1992). Atrial systolic failure in primary amyloidosis. N. Engl. J. Med. 327, 1570–1573. 10.1056/NEJM199211263272205 1435884

[B34] Potse M. Dubé B. Richer J. Vinet A. Gulrajani R. M. (2006). A comparison of monodomain and bidomain reaction-diffusion models for action potential propagation in the human heart. IEEE Trans. Biomed. Eng. 53, 2425–2435. 10.1109/TBME.2006.880875 17153199

[B35] Quarta C. Kruger J. Falk R. (2012). Cardiac amyloidosis. Clin. Med. 126, e178–e182. 10.1161/CIRCULATIONAHA.111.069195 22988049

[B36] Rueckert D. Sonoda L. I. Hayes C. Hill D. L. Leach M. O. Hawkes D. J. (1999). Nonrigid registration using free-form deformations: application to breast MR images. IEEE Trans. Med. Imaging 18, 712–721. 10.1109/42.796284 10534053

[B37] Shlens J. (2014). A Tutorial on Principal Component Analysis.

[B38] Steendijk P. Tulner S. A. Wiemer M. Bleasdale R. A. Bax J. J. van der Wall E. E. (2004). Pressure–volume measurements by conductance catheter during cardiac resynchronization therapy. Eur. Heart J. Suppl. 6(Suppl. D), D35–D42. 10.1016/j.ehjsup.2004.05.012

[B39] Sun J. P. Stewart W. J. Yang X. S. Donnell R. O. Leon A. R. Felner J. M.. (2009). Differentiation of hypertrophic cardiomyopathy and cardiac amyloidosis from other causes of ventricular wall thickening by two-dimensional strain imaging echocardiography. Am. J. Cardiol. 103, 411–415. 10.1016/j.amjcard.2008.09.102 19166699

[B40] Swanton R. H. Brooksby A. I. Davies M. J. Coltart D. J. Jenkins B. S. Webb-Peploe M. M. (1977). Systolic and diastolic ventricular function in cardiac amyloidosis: studies in six cases diagnosed with endomyocardial biopsy. Am. J. Cardiol. 39, 658–664. 10.1016/S0002-9149(77)80125-2 857628

[B41] van der Maaten L. Hinton G. (2008). Visualizing data using t-SNE. JMLR 9, 2579–2605.

[B42] Vogelsberg H. Mahrholdt H. Deluigi C. C. Yilmaz A. Kispert E. M. Greulich S.. (2008). Cardiovascular magnetic resonance in clinically suspected cardiac amyloidosis: noninvasive imaging compared to endomyocardial biopsy. J. Am. Coll. Cardiol. 51, 1022–1030. 10.1016/j.jacc.2007.10.049 18325442

[B43] Wang H. Gao H. Luo X. Berry C. Griffith B. Ogden R.. (2013). Structure-based finite strain modelling of the human left ventricle in diastole. Int. J. Numer. Methods Biomed. Eng. 29, 83–103. 10.1002/cnm.2497 23293070

[B44] Wang H. Luo X. Gao H. Ogden R. Griffith B. Berry C.. (2014). A modified holzapfel-ogden law for a residually stressed finite strain model of the human left ventricle in diastole. Biomech. Model. Mechanobiol. 13, 99–113. 10.1007/s10237-013-0488-x 23609894 PMC3880672

[B45] White J. A. Kim H. W. Shah D. Fine N. Kim K.-Y. Wendell D. C.. (2014). CMR imaging with rapid visual T1 assessment predicts mortality in patients suspected of cardiac amyloidosis. JACC Cardiovasc. Imaging 7, 143–156. 10.1016/j.jcmg.2013.09.019 24412191 PMC3951756

