# Supplementary material for: Analysis of Cardiac Amyloidosis Progression Using Model-Based Markers
Source: Front Physiol. 2020 Apr 30;11:324. doi: 10.3389/fphys.2020.00324 (PMC7203577; doi:10.3389/fphys.2020.00324)
Supplement: Supplementary file 1 [file Data_Sheet_1.pdf]

APPENDIX

A.1 CMR Imaging

Written consents from all patients were acquired before each scan. All participants underwent a standard CMR on a 1. 5T clinical scanner (Aera, Siemens Healthcare, Erlangen, Germany). Retrospectively gated balanced steady state free precession cine images were acquired at both short-axis from base to apex, and long-axis planes including the horizontal long axis, vertical long axis, and left ventricular outflow tract planes, as shown in Fig. A1. Typical imaging parameters for short-axis cine images are: repetition time: 43 ms, echo time: 1.2 ms, field of view: $366 \times 450\,\text{mm}^2$, flip angle: 52°, pixel spacing: $1.4\,$mm, bandwidth: 920 Hz/ pixel, images per cardiac cycle: 25, and slice thickness: 7 mm with a 3 mm slice gap. Late gadolinium enhancement images covering the whole LV were acquired 10-15 minutes after intravenous injection of gadoterate meglumine (gadolinium-DOTA, Dotarem, Guerbet SA, Paris, France) as previously described in (Fontana et al., 2015). The gadolinium dosages for all the scans and cuff-measured pressure during CMR scans are summarized in Table 1.

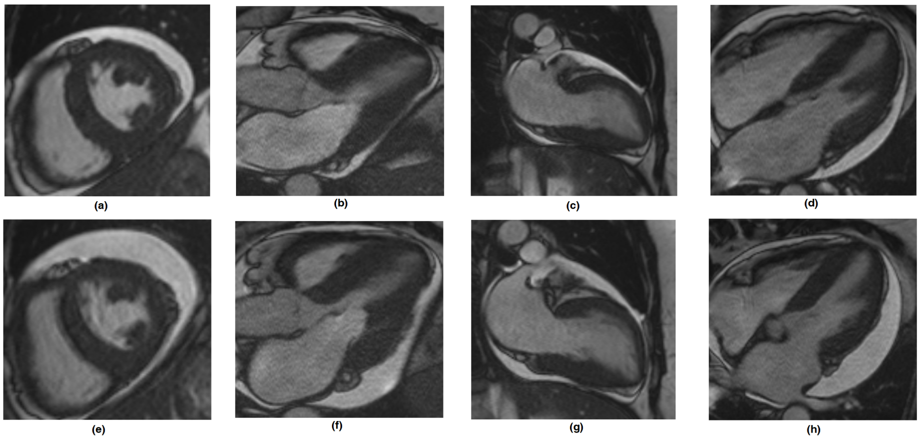

Figure A1. The CMR images that are used to reconstruct LV model in diastole. (a)–(d) are cine images at short-axis and three long-axis planes at the baseline scan, (e)–(h) are corresponding cine images at the follow-up scan from the same patient.

A.2 Two-step optimization, inferred material parameters and Bootstrap evaluation

The flowchart of the two-step optimization method is given in Fig.A2. The inverse problem is solved with the Matlab *lsqnonlin* function using the trust-region-reflective algorithm. The intervals for a, b ,a_{f}, b_{f}, a_{fs} are [0.15, 1], [0.5, 30], [0.2, 30], [0.01, 30], [0.1, 2], respectively, and their initial guesses are generated randomly. The objective function tolerance is chosen to be 10^{-7}, and the relative errors are defined as

$$\begin{cases} \epsilon_V = \sqrt{\frac{1}{n_{\text{time}}}\sum_{i=1}^{n_{\text{time}}}(V_i^{\text{FEA}} - V_i^{\text{CMR}})^2/V_{\text{mean}}^{\text{CMR}^2}} \times 100\% \\ \epsilon_{E\text{cc}} = \sqrt{\frac{1}{n_{\text{layer}}\,n_{\text{reg}}\,n_{\text{time}}}\sum_{k}^{n_{\text{layer}}}\sum_{j=1}^{n_{\text{reg}}}\sum_{i=1}^{n_{\text{time}}}(\bar{E}\text{cc}_{i,j,k}^{\text{FEA}} - \bar{E}\text{cc}_{i,j,k}^{\text{CMR}})^2} \times 100\% \end{cases}. \tag{A1}$$

The optimized material properties are provided in Table A1. The results of a bootstrap analysis of the material parameters of the amyloidosis patients are listed in Table A2, providing parameter estimation uncertainty (Efron and Tibshirani, 1986). We take the circumferential strains obtained from the final

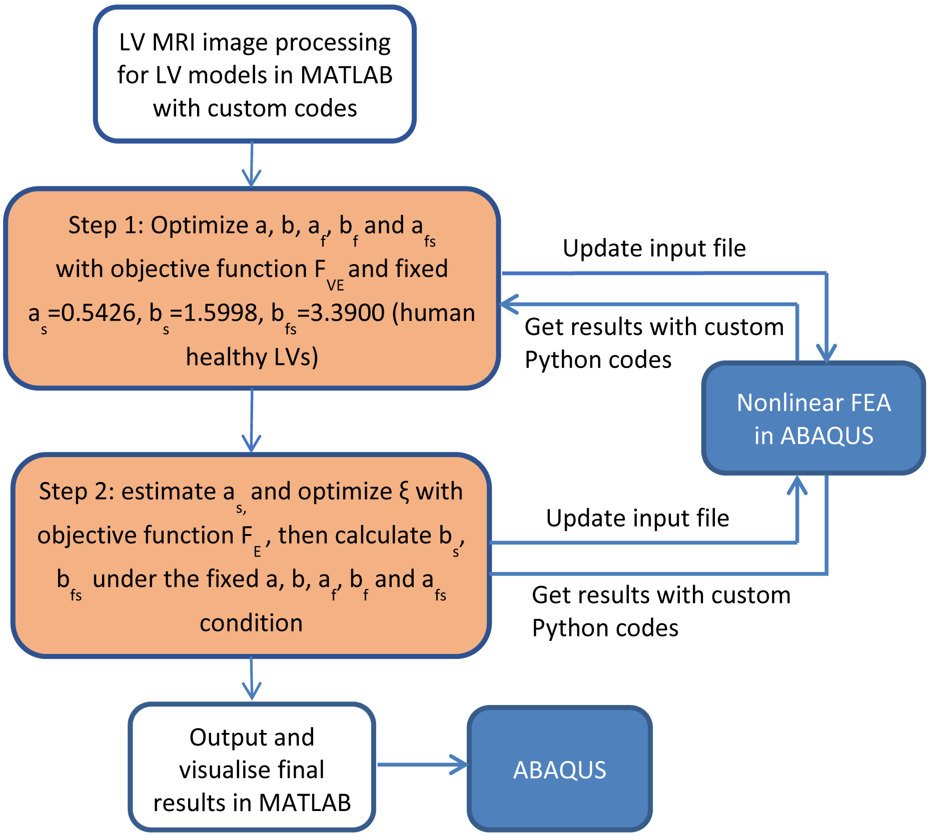

Figure A2. Flow chart of the two-step optimization

parameter estimates, q, and compute the residuals by comparison with the strains extracted from the MRI (i.e. the data to which the original model was fitted). Let us call the set of residuals E. We now generate an ensemble of hypothetical data, $D_1, \ldots, D_K$, by drawing residuals with replacement from E and adding these values to the strains predicted from the model. Note that if we were to add the original residuals to the latter strains, we would recover the original MRI strains. By perturbing the residuals and drawing them with replacement from the set of residuals, we mimick the effect of different noise instantiations. Repeating for $D_1, \ldots, D_K$, the same inference procedure that was performed on the true data, this procedure returns a set of bootstrap estimates, $q_1, \ldots, q_K$, which is summarised by the median and the median absolute deviation (MAD); this is a robust measure of variability that is less susceptible to outliers than the standard deviation. We multiply the MAD with a constant scale factor of k = 1.4826, which makes it a consistent estimator for the estimation of the standard deviation of a normally distributed random variable.

A.3. Dimensionality reduction

Visual representation of the variation in a dataset can be done using the modes of variation. These are obtained by perturbing the mean LV shape along each of the principal components. The top row of figure A3 presents this analysis where the first mode relates to overall size and the second and third modes to the horizontal shape change in the LV. The bottom row of figure A3 presents the amyloidosis patients before treatment projected into PCA space. The first plot shows the first three principal componenets and the

case	stage	a(kPa)	b	a_f (kPa)	b_f	a_fs (kPa)	a_s (kPa)	b_s	b_fs	ε_V, ε_Ecc (%)	R^2 (V, $\mathrm{E_{cc}}$)
1	Baseline	0.32	1.65	4.77	0.18	0.31	1.13	0.10	0.12	2.98, 5.81	0.99, 0.90
	Follow-up	0.58	1.91	2.79	0.14	1.92	1.13	0.05	0.13	3.58, 6.77	0.98, 0.84
2	Baseline	0.20	3.48	1.85	12.66	0.21	0.52	8.78	8.78	3.64, 6.16	0.96, 0.87
	Follow-up	0.67	8.75	0.22	0.57	0.94	0.10	0.31	0.37	4.44, 4.83	0.91 0.89
3	Baseline	0.56	3.09	0.44	0.45	1.16	0.20	0.23	0.30	4.34, 7.85	0.97, 0.84
	Follow-up	0.64	1.40	3.56	0.62	1.39	1.33	0.39	0.41	5.76, 6.79	0.97, 0.85
4	Baseline	0.35	6.18	1.30	11.08	0.19	0.39	5.74	7.25	4.00, 4.80	0.94, 0.86
	Follow-up	0.36	8.10	0.93	9.27	0.52	0.37	4.62	6.08	2.05, 5.82	0.98, 0.85
5	Baseline	0.55	12.85	3.15	14.73	1.46	1.21	4.98	14.06	3.08, 3.23	0.95, 0.88
	Follow-up	0.77	6.69	3.43	1.32	1.04	1.23	0.69	0.86	1.39, 4.12	0.99, 0.87
6	Baseline	0.85	1.51	2.96	0.03	1.34	1.14	0.009	0.02	7.41,16.20	0.95, 0.84
	Follow-up	0.46	1.73	6.49	0.01	1.36	2.15	0.00	0.01	3.58, 6.77	0.97, 0.91
7	Baseline	0.87	4.40	2.99	0.57	1.29	1.14	0.29	0.37	2.66, 4.26	0.98, 0.94
	Follow-up	0.56	2.85	5.65	2.22	0.86	1.73	1.18	1.45	2.50, 6.08	0.99, 0.87

Table A1. Optimized material parameters of the amyloidosis patients. The error ϵ_V varies in (1.39–5.76)%, and the error $\epsilon_{E\mathrm{cc}}$ ranges from 3.23% to 7.85%. The goodness-fit R^2 is listed in the last column for both the volume and strain, respectively.

case	stage	a(kPa)	b	a_f (kPa)	b_f	a_fs (kPa)	ε_V, ε_Ecc (%)
1	Baseline	0.18 (0.02)	1.42 (0.12)	5.22 (0.44)	0.56 (0.15)	1.39 (0.07)	3.86(0.26), 4.92 (0.39)
	Follow-up	0.44 (0.05)	2.05 (0.26)	3.46 (0.21)	0.21 (0.04)	1.06 (0.11)	4.39 (0.06), 5.40 (0.16)
2	Baseline	0.15 (0.002)	0.55 (0.16)	1.66 (0.04)	25.61 (0.78)	0.10 (0.002)	4.43 (0.023), 4.18 (0.17)
	Follow-up	0.52 (0.14)	8.22 (0.42)	0.83 (0.47)	0.17 (0.07)	1.33 (0.10)	6.20 (0.15), 3.69 (0.45)
3	Baseline	0.549 (0.07)	3.16 (0.22)	0.41 (0.06)	0.15 (0.04)	1.25 (0.53)	4.97 (0.03), 3.89 (0.05)
	Follow-up	0.66 (0.06)	1.57 (0.09)	3.81(0.16)	0.28 (0.17)	1.33 (0.20)	8.43 (0.07), 6.05 (0.16)
4	Baseline	0.26 (0.01)	6.18 (0.70)	1.75 (0.43)	12.97 (1.80)	1.06 (0.57)	2.71 (0.07), 3.24 (0.33)
	Follow-up	0.35 (0.07)	7.19 (0.16)	0.97 (0.25)	14.54 (2.95)	0.70 (0.50)	2.86 (0.05) , 4.51 (0.23)
5	Baseline	0.15 (0.002)	14.51 (0.07)	4.93 (0.11)	29.99 (0.02)	1.22 (0.10)	4.88 (0.004), 2.50 (0.10)
	Follow-up	0.80 (0.08)	5.88(0.71)	3.17 (0.38)	1.00 (1.33)	1.95 (0.06)	1.95 (0.06), 2.01 (0.003)
6	Baseline	0.64 (0.10)	1.81 (0.08)	2.79 (0.29)	0.28 (0.23)	1.95 (0.07)	5.43 (0.20), 10.96(0.06)
	Follow-up	0.15 (0.003)	1.71 (0.11)	10.30 (0.20)	0.13 (0.04)	1.96 (0.06)	8.61 (0.07), 4.24 (0.19)
7	Baseline	0.93 (0.10)	4.65 (0.09)	1.76 (0.15)	1.71 (0.22)	1.96 (0.06)	3.40 (0.02) 3.45 (0.13)
	Follow-up	0.65 (0.12)	2.66 (0.35)	5.44 (1.15)	0.89 (1.13)	0.73 (0.03)	3.68 (0.49), 5.91 (0.09)

Table A2. Median (1.4826 $\times$ median absolute deviation) parameter estimates for the amyloidosis patients at baseline and after treatment. Median and MAD volume error and strain error are obtained from the bootstrap parameter estimates.

second shows the second PC which appears to relate to wall thickness. The final plot provides projection using t-SNE where we observe a clear clustering of the amyloidosis patients.

A.4 Values of the biomarkers at the baseline and follow-up

Here we compute, for each of the parameter estimates $q_1, \ldots, q_K$ from the bootstrap analysis in turn, the corresponding derived quantities from Table 4 in the paper. Results are summarized by the median and MAD estimates from these bootstrap samples in Table A3.

The results in Table A4 are used for the uncertainty quantification of the recovery scores in Table 4. This is done as follows. The scores in Table 4 are of the form

$$X = \frac{F - B}{B} = \frac{F}{B} - 1 \tag{A2}$$

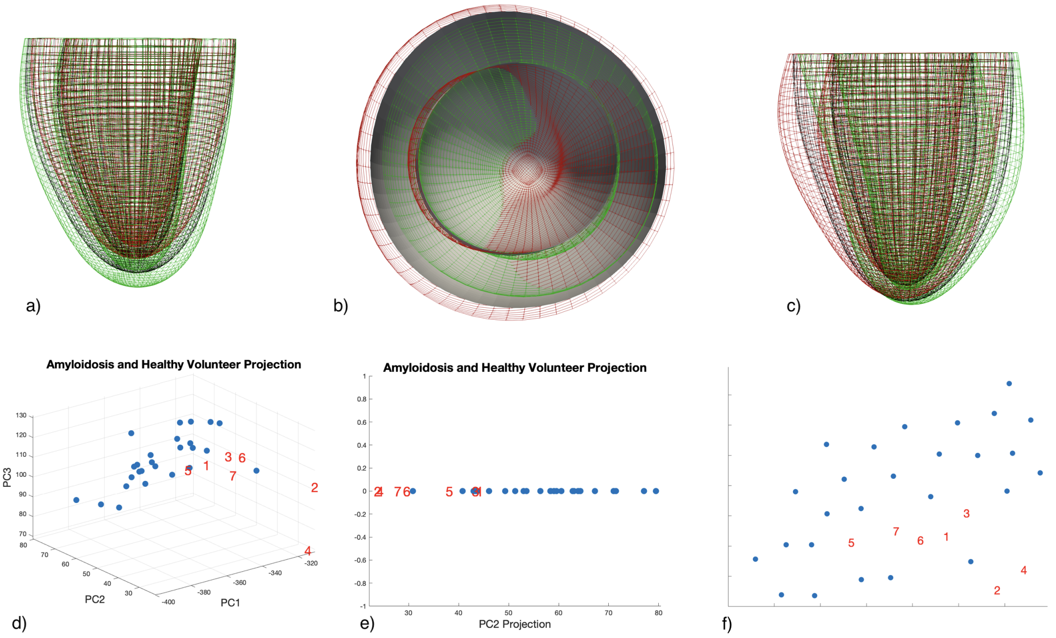

Figure A3. *Modes 1, 2 and 3 obtained using PCA are shown in (a) (b) and (c), respectively. For modes 1 and 3, the mean shape is black, green subtracts a shape along the principal component (PC) and red adds a shape along the PC. For mode 2, the mean shape is given by a solid grey shape for ease of representation. (d) and (e) show the projections onto the first three and the second PC where red numbers indicate an Amyloidosis patient before treatment and blue points are healthy volunteers. (c) gives the projection obtained using tSNE.*

case	stage	$\bar{E}_{\mathrm{cc}}$	$\bar{E}_{\mathrm{ll}}$	$\bar{\sigma}_1$	W	f
1	Baseline	0.27 (0.004)	0.32 (0.012)	8.79 (0.166)	93.28(2.518)	34.13 (1.821)
	Follow-up	0.31 (0.007)	0.29 (0.01)	7.21 (0.16)	78.51 (4.64)	21.99 (1.75)
2	Baseline	0.14 (0.001)	0.20 (0.001)	6.36 (0.01)	18.27 (0.17)	196.68 (11.43)
	Follow-up	0.18 (0.007)	0.10 (0.012)	3.89 (0.082)	18.26 (1.651)	9.34(9.645)
3	Baseline	0.34 (0.004)	0.18(0.007)	5.97 (0.038)	56.04 (3.198)	6.42 (1.003)
	Follow-up	0.32 (0.004)	0.30 (0.008)	8.37 (0.181)	153.15 (1.017)	35.60 (4.669)
4	Baseline	0.16 (0.006)	0.17 (0.007)	5.67 (0.22)	29.41 (1.23)	66.49 (23.31)
	Follow-up	0.167 (0.005)	0.15 (0.007)	5.34 (0.228)	20.26 (0.665)	39.33 (18.14)
5	Baseline	0.09 (0.0003)	0.10 (0.0006)	4.93 (0.029)	22.89 (0.148)	372.90 (12.862)
	Follow-up	0.16 (0.008)	0.10 (0.009)	4.42 (0.218)	47.58 (0.784)	25.48 (14.321)
6	Baseline	0.28 (0.004)	0.26 (0.008)	6.71 (0.118)	121.21 (2.200)	20.03 (0.974)
	Follow-up	0.20 (0.003)	0.26 (0.001)	7.59 (0.059)	103.22 (0.870)	57.69 (14.996)
7	Baseline	0.21 (0.002)	0.11 (0.002)	5.41 (0.026)	60.30 (0.461)	18.83 (0.973)
	Follow-up	0.23 (0.008)	0.20 (0.007)	7.69(0.412)	153.09 (5.048)	51.02(22.921)

Table A3. Median (1.4826 $\times$ median absolute deviation) biomarkers for the amyloidosis patients at baseline and after treatment.

where B stands for baseline and F for follow-up. By the standard Gaussian error propagation rule, the variance of X is:

$$\sigma_X^2 = \sqrt{\left(\frac{\partial X}{\partial F}\right)^2 \sigma_F^2 + \left(\frac{\partial X}{\partial B}\right)^2 \sigma_B^2} = \sqrt{\frac{\sigma_F^2}{B^2} + \frac{F^2}{B^4}\sigma_B^2} \tag{A3}$$

where we replace the variances, σ_F^2 and σ_B^2, by the squared adjusted MADs as their robust equivalent.

REFERENCES

Efron, B. and Tibshirani, R. (1986). Bootstrap methods for standard errors, confidence intervals, and other measures of statistical accuracy. *Statistical science* , 54–75

Fontana, M., Pica, S., Reant, P., Abdel-Gadir, A., Treibel, T. A., Banypersad, S. M., et al. (2015). Prognostic value of late gadolinium enhancement cardiovascular magnetic resonance in cardiac amyloidosis. *Circulation* 132, 1570–1579
