# Supplementary figures and images for: Analysis of Cardiac Amyloidosis Progression Using Model-Based Markers
Source: Front Physiol. 2020 Apr 30;11:324. doi: 10.3389/fphys.2020.00324 (PMC7203577; doi:10.3389/fphys.2020.00324)

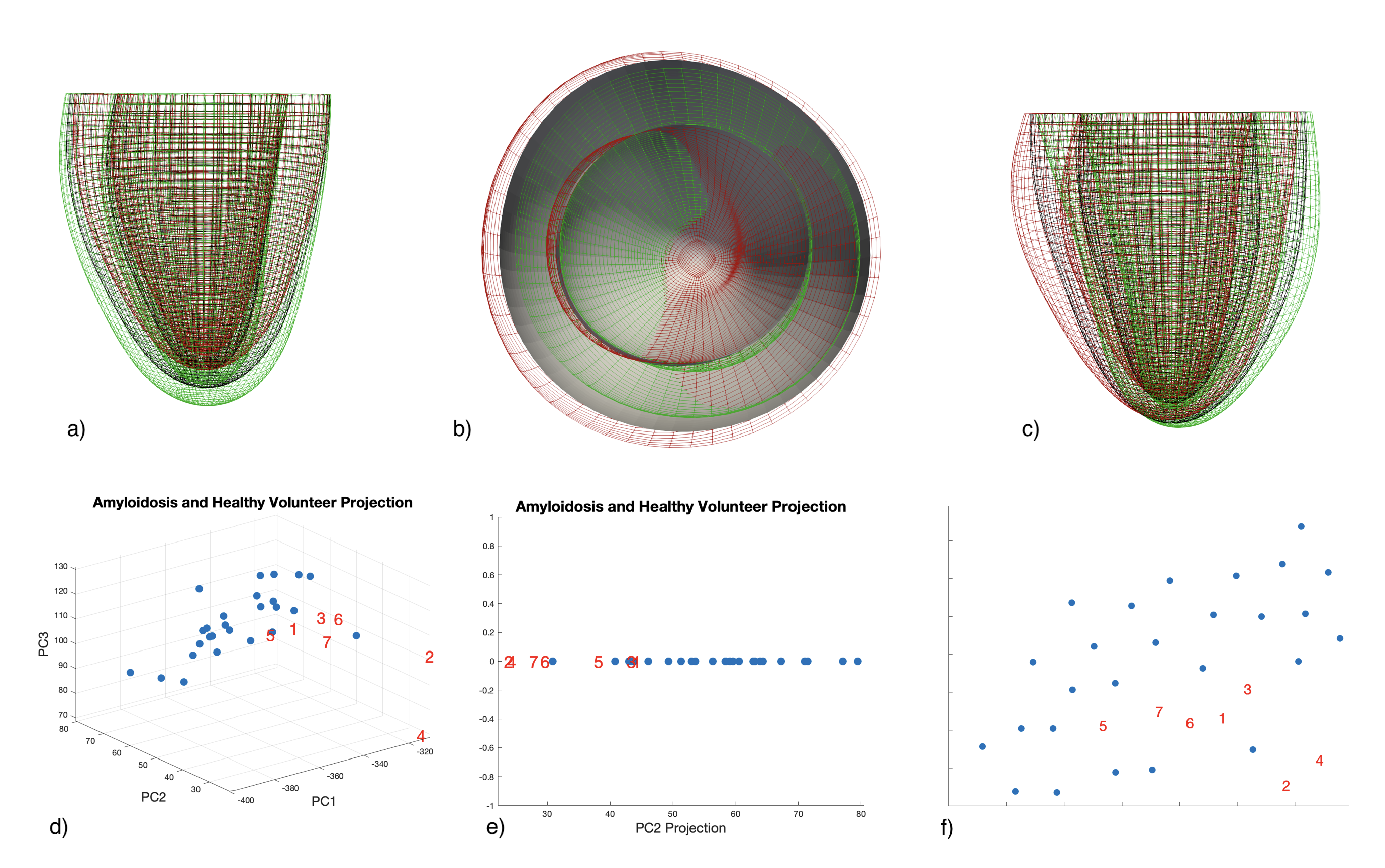

Supplement: Supplementary file 2 [file Data_Sheet_2.zip › pca_modes.png]

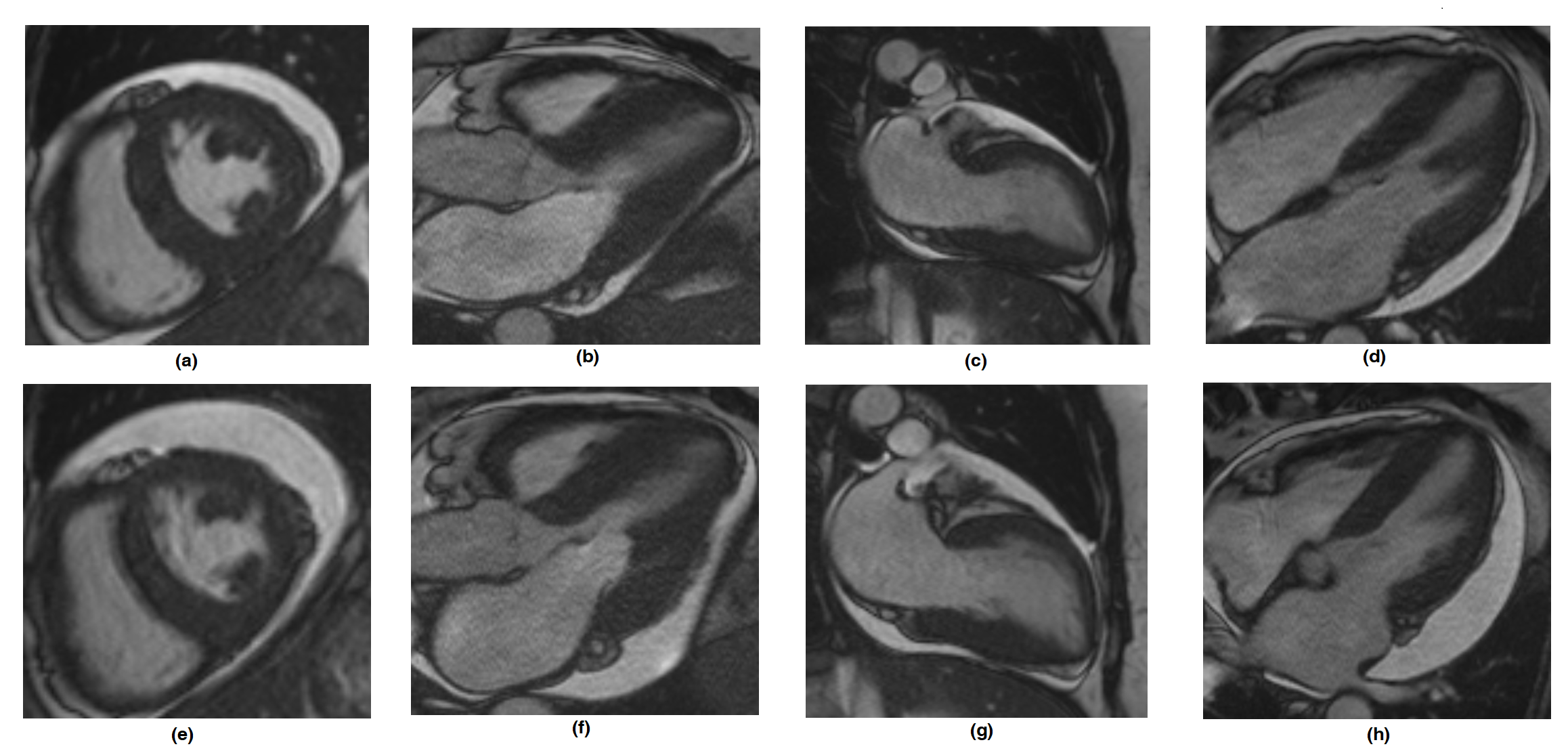

Supplement: Supplementary file 2 [file Data_Sheet_2.zip › fig1_CMRImages.png]

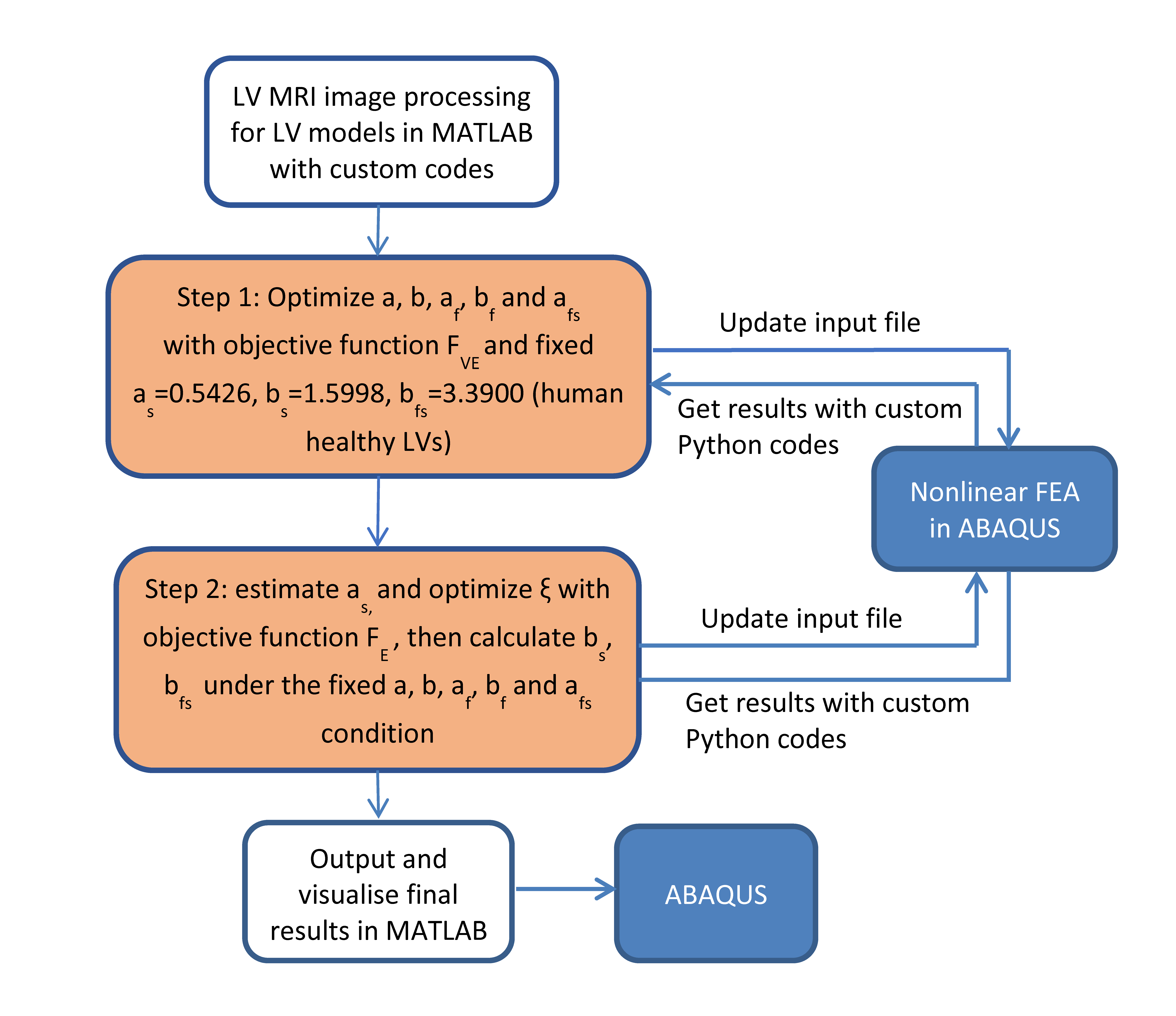

Supplement: Supplementary file 2 [file Data_Sheet_2.zip › optimization_steps.png]
